# Predicting extubation in patients with traumatic cervical spinal cord injury using the diaphragm electrical activity during a single maximal maneuver

**DOI:** 10.1186/s13613-023-01217-7

**Published:** 2023-12-06

**Authors:** Rui Zhang, Xiaoting Xu, Hui Chen, Jennifer Beck, Christer Sinderby, Haibo Qiu, Yi Yang, Ling Liu

**Affiliations:** 1 Jiangsu Provincial Key Laboratory of Critical Care Medicine, Department of Critical Care Medicine, Zhongda Hospital, School of Medicine,, Southeast University, Nanjing, 210009 Jiangsu China; 2 Department of Critical Care Medicine, The First Affiliated Hospital of Soochow University, Soochow University, No. 899 Pinghai Road, Suzhou, 215000 People’s Republic of China; 3 Keenan Research Centre for Biomedical Science of St. Michael’s Hospital, Department of Critical Care, St. Michael’s Hospital, Toronto, Canada; 4 Department of Pediatrics, University of Toronto, Toronto, Canada; 5 Member, Institute for Biomedical Engineering and Science Technology (iBEST) at Ryerson University and St-Michael’s Hospital, Toronto, Canada; 6 Department of Medicine and Interdepartmental Division of Critical Care Medicine, University of Toronto, Toronto, Canada

**Keywords:** Cervical spinal cord injury, Diaphragm electrical activity, Single maximal maneuver, Extubation

## Abstract

**Background:**

The unsuccessful extubation in patients with traumatic cervical spinal cord injuries (CSCI) may result from impairment diaphragm function and monitoring of diaphragm electrical activity (EAdi) can be informative in guiding extubation. We aimed to evaluate whether the change of EAdi during a single maximal maneuver can predict extubation outcomes in CSCI patients.

**Methods:**

This is a retrospective study of CSCI patients requiring mechanical ventilation in the ICU of a tertiary hospital. A single maximal maneuver was performed by asking each patient to inhale with maximum strength during the first spontaneous breathing trial (SBT). The baseline (during SBT before maximal maneuver), maximum (during the single maximal maneuver), and the increase of EAdi (ΔEAdi, equal to the difference between baseline and maximal) were measured. The primary outcome was extubation success, defined as no reintubation after the first extubation and no tracheostomy before any extubation during the ICU stay.

**Results:**

Among 107 patients enrolled, 50 (46.7%) were extubated successfully at the first SBT. Baseline EAdi, maximum EAdi, and ΔEAdi were significantly higher, and the rapid shallow breathing index was lower in patients who were extubated successfully than in those who failed. By multivariable logistic analysis, ΔEAdi was independently associated with successful extubation (OR 2.03, 95% CI 1.52–3.17). ΔEAdi demonstrated high diagnostic accuracy in predicting extubation success with an AUROC 0.978 (95% CI 0.941–0.995), and the cut-off value was 7.0 μV.

**Conclusions:**

The increase of EAdi from baseline SBT during a single maximal maneuver is associated with successful extubation and can help guide extubation in CSCI patients.

**Supplementary Information:**

The online version contains supplementary material available at 10.1186/s13613-023-01217-7.

## Introduction

Patients with traumatic cervical spinal cord injury (CSCI) typically require mechanical ventilation due to complete or partial denervation of the diaphragm and intercostal muscles, paralysis of the thoracic and abdominal walls during their acute admission [1, 2]. Tracheostomy is preferred when prolonged mechanical ventilation is anticipated [3]; however, about one-third to two-thirds of patients with respiratory failure following CSCI can be extubated and weaned successfully during acute hospitalization [4–7]. Given that an early primary tracheostomy, as opposed to a secondary tracheostomy following extubation failure or late tracheostomy, may decrease ICU mortality and length of stay [8, 9], it is essential to accurately distinguish between CSCI patients who can be extubated and those who require long-term mechanical ventilation and will perhaps benefit from tracheotomy. Although many studies have investigated the risk factors for extubation failure [5, 10] and early tracheostomy after CSCI [11–14], the results are debatable. How to precisely predict the extubation outcome of CSCI patients and when to perform tracheostomy are still challenging.

Factors associated with neural pathways, such as the American Spinal Injury Association (ASIA) impairment scale A grade or B grade and high neurological level of injury, were crucial in determining extubation outcome and tracheostomy performance [10, 14]. Injuries above the level of phrenic motor neurons (primarily located at *C*3–*C*5) can cause both diaphragm and expiratory muscle paralysis, leading to inspiratory failure and inadequate clearance of secretions, resulting in extubation failure. Since the diaphragm is the main muscle involved in inspiration [3], the disruption of innervation to the diaphragm may be critical in determining whether patients with CSCI can be extubated successfully. It has been found that negative inspiration force generated by surrogate measurements of needle electromyography (EMG) of the diaphragm best predicted the ability to wean from the ventilator in CSCI patients, compared with the fluoroscopic examination of the diaphragm, bedside spirometry, and ASIA scales [15].

However, the needle EMG is invasive, cannot be continuously monitored, and is prone to artifacts, such as cross-talk from other muscles. When the patient uses the diaphragm as the primary respiratory muscle with intactness of phrenic nerve conduction, and assuming that the crural diaphragm activity can represent the total diaphragm activity, esophageal recordings of diaphragm electrical activity (EAdi) can reflect the respiratory drive [16]. As a substitute and optimization of needle EMG, EAdi make it possible to continuously and less invasively monitor the respiratory neuromuscular function. EAdi can be used to monitor changes in respiratory drive and effort and has provided valuable information during spontaneous breathing trial (SBT) for evaluating extubation readiness [17, 18]. Herein, we designed a “single maximal maneuver” test to monitor the maximum EAdi during the maximal inspiration in CSCI patients during SBT. The EAdi monitoring and the change of EAdi during forced breathing could indicate the extent of phrenic nerve injury and the preservation of neuromuscular function. We calculate ΔEAdi as the difference between the maximum EAdi during a simple “single maximal maneuver” and the EAdi during SBT. The study aimed to investigate the association between ΔEAdi and extubation outcomes in CSCI patients and further evaluate whether ΔEAdi can predict extubation success.

## Methods

### Patients

This retrospective cohort study enrolled traumatic CSCI patients who underwent mechanical ventilation and were admitted to the intensive care unit (ICU) of Zhongda Hospital, Southeast University, Nanjing, China, from June 2014 to April 2023. Inclusion criteria were: (1) age 18 years or older; (2) traumatic CSCI patients with a neurologic level of injury of *C*1 to *C*8 by the ASIA standard impairment scale grade A to D [19]; (3) with invasive mechanical ventilation due to acute respiratory failure; (4) with a dedicated nasogastric tube with nine electrodes that allow to continuously measure EAdi (EAdi catheter, Maquet, Solna, Sweden) in position. CSCI was defined as a radiologically confirmed injury to the cervical spinal column, combined with the clinical signs and symptoms consistent with that level.

The exclusion criteria were: (1) tracheostomy before ICU admission; (2) withhold or withdraw life-sustaining treatment due to other severe organ injuries; (3) cannot tolerate any SBT or complete instructional actions; (4) death occurred within seven days after injury; (5) postoperative mechanical ventilation without respiratory failure and had a ventilation duration of fewer than 24 h postoperatively; (6) no EAdi wave.

### Single maximal maneuver

According to the local hospital protocol, the standard nasogastric tubes of CSCI patients were replaced by a dedicated 16-F nasogastric tube with nine electrodes that allow for the measurement of EAdi on the first day of ICU admission. All enrolled patients were ventil**a**ted with a Servo-I ventilator (Maquet, Solna, Sweden; software version 4.01). “Single maximal maneuver” was performed during the SBT to assist the decision for extubation and avoid delayed weaning. The sedative drugs were discontinued or retained at low doses to avoid accidental catheter dislocation. A 30-min SBT was conducted with continuous positive airway pressure (CPAP) of 5 cmH_2_O at the prescribed inspired fraction of oxygen and followed by a blood gas analysis. Baseline EAdi, tidal volume (VT), and rapid shallow breathing index (RSBI) were recorded during CPAP. Baseline EAdi and VT were calculated as the mean value of the five consecutive stable breaths. Baseline neuroventilatory efficiency (NVE) was calculated as the ratio of VT and EAdi during inspiration [18]. When the patient can tolerate the CPAP for the initial three minutes, the “single maximal maneuver” was performed by asking the patient to breathe with maximum strength without airway occlusion (twice at 30 s intervals), and the maximum inspiratory EAdi (Max EAdi) and the maximum VT (Max VT) were recorded (average value of the two breaths). EAdi-derived parameters were calculated as follows: relative activation of the diaphragm at baseline (Rel EAdi) = baseline EAdi/Max EAdi × 100%, an absolute increase of EAdi (ΔEAdi) = Max EAdi–baseline EAdi, and relative increase of EAdi (ΔEAdi%) = (Max EAdi–baseline EAdi)/baseline EAdi × 100%.

During the SBT, patients should be switched to the previous ventilation mode when they met the following criteria: (1) tachypnea with respiratory rate > 40 breaths/min; (2) hypoxemia with SpO_2_ < 90%; (3) hypercapnia with end-tidal carbon dioxide pressure increased by more than 20 mmHg, or consciousness deterioration or agitation; (4) hemodynamic instability with systolic blood pressure (SBP) < 90 mmHg, or 60 beats/min > heart rate > 140 beats/min, or severe arrhythmias; (5) other conditions by attending physician’s judgment.

### Local weaning protocol

All CSCI patients were screened daily (between 8:00 and 10:00 am) by the physician in charge to assess the possibility of the SBT according to the local protocol. Patients who could succeed in the 30-min SBT and cuff leak test and demonstrated adequate cough ability were extubated. The online supplement gives more details about the screening for eligibility of SBT, criteria for SBT failure, assessment for cuff leak test and cough strength, local protocols of post-extubation noninvasive respiratory support, and reintubation criteria. The physician in charge decided on the timing of tracheostomy in patients who could not tolerate the SBT or failed the first extubation.

### Measurements and data collection

The following data were extracted from the local trauma center (Level 2) registries and the ICU clinical information system: demographics, mechanism of injury, associated injuries, comorbidities, Injury Severity Score (ISS), cervical operation, level of CSCI, ASIA classification, and AIS score on the day of ICU admission. The Acute Physiology and Chronic Health Evaluation (APACHE) II score and Sequential Organ Failure Assessment (SOFA) score were assessed during the first 24 h of ICU admission. Glasgow Coma Score (GCS), Richmond Agitation-Sedation Scale (RASS), breathing pattern, and blood gas analysis were recorded during SBT. Clinical outcomes were recorded, including extubation and weaning outcomes, the incidence of ventilator-associated pneumonia (VAP), total duration of mechanical ventilation, ventilator-free days within 7, 14, and 28 days after mechanical ventilation, ICU and hospital length of stay, and ICU and hospital mortality.

The primary outcome was first extubation success, defined as no need for reintubation after the first extubation and no tracheostomy before any extubation during the ICU stay. Patients who were reintubated, tracheostomized or died before ICU discharge without extubation were classified into unsuccessful extubation group. Secondary outcomes included weaning success, defined as no ventilatory support for more than 48 consecutive hours or transfer from ICU without the ventilator, ICU and hospital mortality, ventilator-associated pneumonia incidence, ICU and hospital length of stay. The trial was registered at ClinicalTrials. gov (NCT04089956) and approved by the Institutional Ethics Committee of Zhongda Hospital (2022ZDSYLL337-P01). All data were kept confidential, and written informed consent was waived due to the retrospective observational nature.

### Statistical analysis

Continuous data were reported as mean (standard deviation, SD) or median (interquartile range [IQR]), and categorical data as the number of events (percentages). Differences between groups were assessed with the *t*-test or Mann–Whitney test for continuous variables and the Chi test for categorical variables. All statistical analyses were done using MedCalc (version 20.0.3) and RStudio (version 1.3.1073). Two-tailed *p *< 0.05 was considered statistically significant.

We first evaluated the association between ΔEAdi and extubation outcomes using the multivariable logistic regression model. Based on published studies, clinical relevance [5, 20], and given the principle of ten events per variable [21], variables including SOFA, RSBI, high cervical spinal cord injury and PaO_2_/FiO_2_ were selected for the multivariable analysis. The variance inflation factor (VIF) method was used to examine the colinearity, and variables with VIF ≥ 5 suggested multicolinearity (details in Additional file 1).

Then, we assessed the accuracy of ΔEAdi for classifying patients who would succeed extubation. Receiver operating characteristic (ROC) curve was performed, and the area under ROC curve (AUROC) was calculated. We also calculated the AUROC of ΔEAdi after adjustment for aforementioned variables. Considering existing studies have revealed predictive value of RSBI, NVE, tidal volume for successful extubation [18, 20], we compared the predictive performance of ΔEAdi and these variables. Bootstrap estimated the 95% confidence interval of AUROC and the Delong test was used to compare AUROCs of different variables. The optimal threshold of continuous variables was chosen to maximize the Youden index. We further calculated the net reclassification index (NRI) to explore whether the ΔEAdi improved successful extubation classification over other parameters. Decision curve analysis was conducted by quantifying the net benefits at different threshold probabilities.

## Results

### Characteristics of the enrolled population

**Fig. 1:**
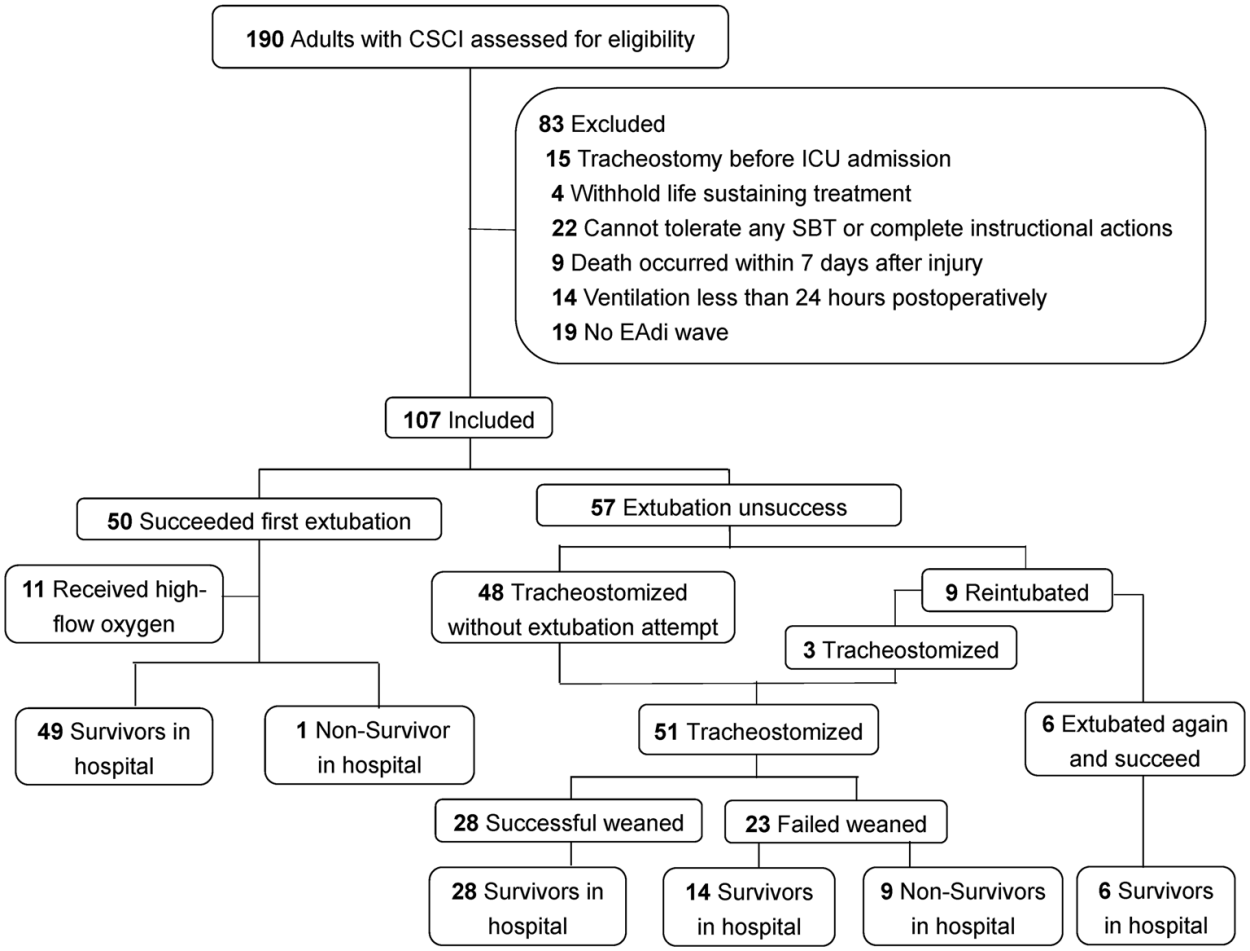
Flowchart of the study. CSCI, cervical spinal cord injury; ICU, intensive care unit; EAdi, diaphragm electrical activity

**Fig. 2:**
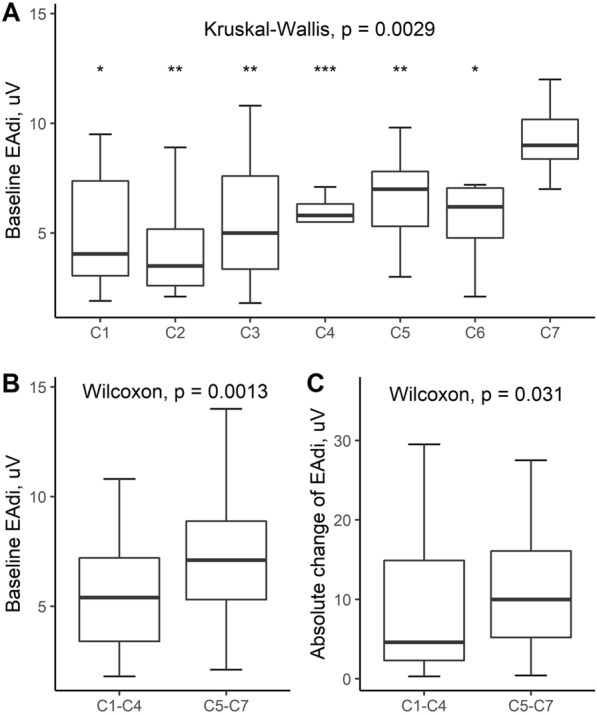
Boxplot showing the distribution of **A** baseline EAdi in patients ranged from *C*1–*C*7, compared with *C*7, *p < 0.05, **p < 0.01, ***p < 0.001. **B** *C*1–*C*4 vs. *C*5–*C*7, and (**C**) absolute change of EAdi in *C*1–*C*4 vs. *C*5–*C*7

Among 190 adult CSCI patients receiving invasive mechanical ventilation during the study, 107 were enrolled in the final analysis (Fig. 1). Of the 107 cases, the average age was 60 (14) years, and 89 (83.2%) were male. More than half of the patients suffered from high (*C*1–*C*4) CSCI, and 44.9% had complete motor injury (ASIA grade of A and B). Both baseline EAdi and the ΔEAdi during the single maximal maneuver were lower in patients with high CSCI (Fig. 2) and complete motor injury (Additional file 1: Table S2). 99 (92.5%) patients underwent surgical intervention for fixation of cervical spinal column and decompression of spinal cord. Fifty (46.7%) patients succeeded in the first extubation, and 11 received post-extubation high-flow nasal oxygen therapy. Of the 57 (53.3%) unsuccessfully extubated patients, 48 were tracheostomized without extubation, and nine were reintubated. Overall, 51 (47.7%) patients underwent tracheostomy after 6 [3, 9] days of initializing invasive mechanical ventilation, and 84 (78.5%) patients were weaned from ventilator. The plausible clinical reasons for the unsuccessful extubation included impaired cough efficacy (29/57), followed by SBT failure (19/57) during extubation screening, and post-extubation respiratory failure (9/57). Eight patients died in the ICU, and ten patients died during hospitalization.

### Comparisons between successful and unsuccessful extubation patients

Patients in the successful and unsuccessful extubation group were balanced at baseline regarding sex, age, RASS, mechanism of cervical injury, associated injuries, and comorbidities (Table 1 and Additional file 1: Table S1). Compared with the extubation unsuccess group, patients who succeeded in the first extubation were generally less severe, as indicated by the lower APACHE II, SOFA score, and ISS, in addition to the lower incidence of the high neurologic level of injury and complete motor dysfunction.

**Table 1:** Demographic and clinical characteristics of included patients

	Overall (*n* = 107)	Successful extubation (*n* = 50)	Unsuccessful extubation (*n* = 57)	*p* value
Age, year	60 (14)	59 (13)	60 (15)	0.866
Male, *n* (%)	89 (83.2)	39 (78.0)	50 (87.7)	0.279
Body mass index	23.9 [22.8, 25.9]	24.2 [22.9, 26.6]	23.5 [22.7, 25.4]	0.230
APACHE II	13 [9, 18]	10 [8, 14]	15 [11, 20]	0.001
SOFA	5 [3, 7]	4 [2, 6]	6 [4, 8]	< 0.001
ISS	17 [14, 25]	16 [11, 18]	21 [16, 25]	0.001
GCS	15 [13, 15]	15 [14, 15]	15 [12, 15]	0.019
Richmond Agitation-Sedation Scale				0.181
− 1	11 (10.3)	3 (6.0)	8 (14.0)	
0	67 (62.6)	30 (60.0)	37 (64.9)	
1	29 (27.1)	17 (34.0)	12 (21.1)	
C-spine injury level, *n* (%)				0.035
*C*1	6 (5.6)	2 (4.0)	4 (7.0)	
*C*2	10 (9.3)	1 (2.0)	9 (15.8)	
*C*3	31 (29.0)	12 (24.0)	19 (33.3)	
*C*4	18 (16.8)	9 (18.0)	9 (15.8)	
*C*5	18 (16.8)	12 (24.0)	6 (10.5)	
*C*6	12 (11.2)	5 (10.0)	7 (12.3)	
*C*7	12 (11.2)	9 (18.0)	3 (5.3)	
*C*1–*C*4, *n* (%)	65 (60.7)	24 (48.0)	41 (71.9)	0.020
ASIA grade, *n* (%)				< 0.001
*A*	16 (15.0)	5 (10.0)	11 (19.3)	
*B*	32 (29.9)	7 (14.0)	25 (43.9)	
*C*	49 (45.8)	28 (56.0)	21 (36.8)	
*D*	10 (9.3)	10 (20.0)	0 (0.0)	
ASIA *A* + *B*, *n* (%)	48 (44.9)	12 (24.0)	36 (63.2)	< 0.001
Cause of injury, *n* (%)				0.563
Falling	42 (39.3)	17 (34.0)	25 (43.9)	
Motor vehicle collision	52 (48.6)	26 (52.0)	26 (45.6)	
Other	13 (21.1)	7 (14.0)	6 (10.5)	
Surgical intervention, *n* (%)	99 (92.5)	49 (98.0)	50 (87.7)	0.099
Comorbidity, *n* (%)				
Cardiovascular system	39 (36.4)	17 (34.0)	22 (38.6)	0.771
Respiratory system	0 (0.0)	0 (0.0)	0 (0.0)	−
Neurological system	9 (8.4)	3 (6.0)	6 (10.5)	0.622
Endocrine system	14 (13.1)	5 (10.0)	9 (15.8)	0.549
Digestive system	5 (4.7)	4 (8.0)	1 (1.8)	0.285
Immune dysfunction	5 (4.7)	2 (4.0)	3 (5.3)	1.000

APACHE, Acute Physiology and Chronic Health Evaluation; ASIA, American Spinal Injury Association; ISS, injury severity score; GCS, Glasgow Coma Score; SOFA, Sequential Organ Failure Assessment

**Fig. 3:**
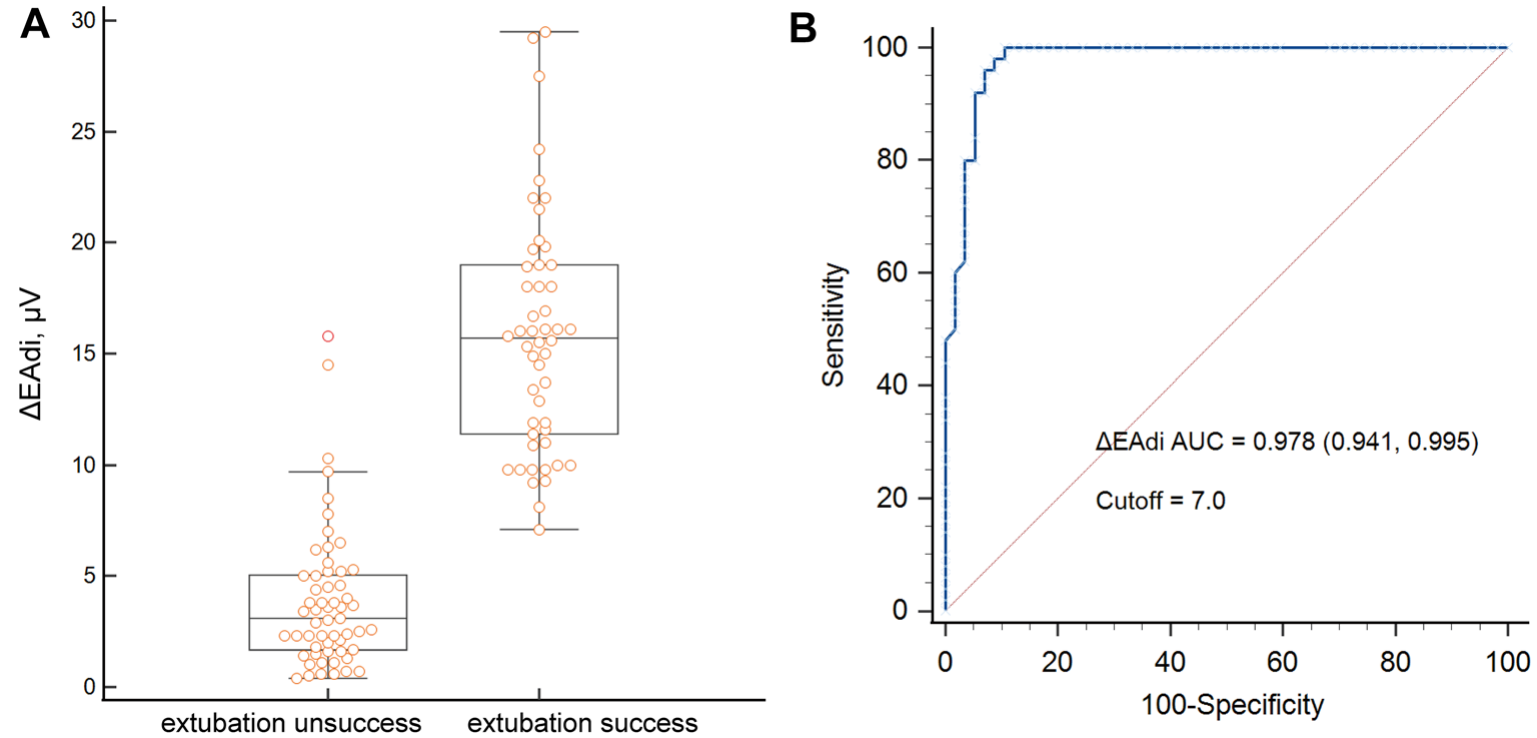
The performance of ΔEAdi to predict extubation outcomes. **A** comparison of ΔEAdi between the successful and unsuccessful extubation group; **B** receiver operating curve for ΔEAdi to predict extubation success

Table 2 shows the respiratory parameters during SBT and the “single maximal maneuver.” In patients who were extubated successfully, the EAdi and tidal volume, both at baseline and the maximal, were significantly higher, and the ΔEAdi was also higher (Fig. 3A), while respiratory rate, RSBI, and PaCO_2_ were substantially lower than those who failed extubation. It is worth noting that the relative activation of the diaphragm during SBT, expressed as the ratio of baseline EAdi to the maximum EAdi (Rel EAdi%), the baseline and maximal NVE were higher in the unsuccessful extubation group.

**Table 2:** Comparisons of respiratory variables during spontaneous breathing trial and the maximal maneuver between extubation success and failure group

	Overall (*n* = 107)	Successful extubation (*n* = 50)	Unsuccessful extubation (*n* = 57)	*p* value
Respiratory rate, bpm	27 (6)	24 (5)	29 (6)	< 0.001
Baseline VT, mL/kg	4.6 [3.3, 5.5]	5.3 [4.6, 5.7]	3.3 [2.8, 5.1]	< 0.001
Maximal VT, mL/kg	9.3 [4.9, 11.4]	11.1 [9.7, 14.1]	5.0 [3.7, 8.9]	< 0.001
ΔVT, mL/kg	4.1 [1.7, 6.2]	6.2 [4.8, 8.7]	1.7 [1.1, 3.6]	< 0.001
RSBI	88 [62, 140]	72 [58, 88]	137 [78, 178]	< 0.001
Baseline EAdi, μV	5.9 [4.2, 8.0]	7.8 [6.2, 9.4]	4.3 [3.1, 5.7]	< 0.001
Maximal EAdi, μV	14.8 [6.8, 22.5]	22.5 [19.8, 27.8]	7.5 [4.6, 10.5]	< 0.001
Rel EAdi, %	48.5 [33.9, 58.8]	32.7 [27.7, 39.9]	57.9 [53.2, 65.6]	< 0.001
ΔEAdi, μV	8.1 [3.0, 15.6]	15.7 [11.5, 19.0]	3.1 [1.6, 5.0]	< 0.001
ΔEAdi, %	110 [70, 190]	210 [150, 260]	70 [50, 90]	< 0.001
Baseline NVE, mL/μV	51.6 [40.5, 67.1]	44.1 [35.9, 52.9]	60.6 [46.4, 74.1]	< 0.001
Maximal NVE, mL/μV	40.0 [31.5, 58.7]	32.0 [28.3, 36.5]	55.2 [41.9, 66.5]	< 0.001
pH	7.43 [7.41, 7.45]	7.43 [7.41, 7.46]	7.43 [7.41, 7.45]	0.913
PaCO_2_, mmHg	36.2 [32.1, 39.8]	33.5 [30.7, 37.5]	37.8 [34.9, 41.0]	0.001
PaO_2_/FiO_2_, mmHg	306.6 [248.3, 366.2]	335.5 [285.1, 386.5]	280.0 [220.8, 349.8]	< 0.001
Bicarbonate, mmol/L	24.0 [22.2, 26.5]	23.0 [20.8, 24.7]	25.9 [23.5, 27.6]	< 0.001
Lac, mmol/L	1.5 [1.1, 2.2]	1.5 [0.9, 2.2]	1.6 [1.1, 2.2]	0.559

EAdi, diaphragm electrical activity; Rel EAdi, relative activation of diaphragm at baseline; FiO_2_, fraction of inspired oxygen; NVE, neuroventilatory efficiency; RSBI, rapid shallow breathing index; VT, tidal volume normalized to predicted body weight; PaCO_2_, partial pressure of arterial carbon dioxide; PaO_2_, partial pressure of arterial oxygen

Table 3 shows that, compared to the unsuccessful extubation group, patients who succeeded in the extubation had a shorter total mechanical ventilation duration and length of stay in the ICU and hospital. The incidence of ventilator-associated pneumonia and hospital mortality was also lower in the extubation success group.

**Table 3:** Clinical outcomes of included patients

	Overall (*n* = 107)	Successful extubation (*n* = 50)	Unsuccessful extubation (*n* = 57)	*p* value
Weaning success, *n* (%)	84 (78.5)	50 (100.0)	34 (59.6)	< 0.001
Patients tracheotomized, *n* (%)	51 (47.7)	0 (0.0)	51 (89.5)	< 0.001
Time to tracheostomy, days	6 [3, 9]	–	6 [3, 9]	–
Duration of MV, days	5 [2, 18]	2 [1, 2]	16 [9, 30]	< 0.001
VFDs by day 7, days	1 [0, 5]	5 [5, 6]	0 [0, 0]	< 0.001
VFDs by day 14, days	8 [0, 12]	12 [12, 13]	0 [0, 0]	< 0.001
VFDs by day 28, days	22 [1, 26]	26 [26, 27]	4 [0, 14]	< 0.001
Incidence of VAP, *n* (%)	23 (21.5)	4 (8.0)	19 (33.3)	0.003
Hospital survival, *n* (%)	97 (90.7)	49 (98.0)	46 (80.7)	0.035
LOS of ICU, days	10 [3, 22]	3 [1, 6]	19 [12, 30]	< 0.001
LOS of hospital, days	19 [13, 31]	15 [12, 24]	25 [15, 35]	0.010

ICU, intensive care unit; LOS, length of stay; MV, mechanical ventilation; VFD, ventilator-free day; VAP, ventilator-associated pneumonia;

### Diagnostic performance of ΔEAdi in prediction of extubation success

The multivariable analysis (Additional file 1: Table S4) reveals that the higher ΔEAdi (OR 2.03, 95% CI 1.52–3.17, *p* < 0.001) during the single maximal maneuver was independently associated with the higher likelihood of successful extubation. ΔEAdi during single maximal maneuvers demonstrated high discriminative power for the prediction of extubation success, and the AUROC was 0.978 (95% CI 0.941–0.995). With a cut-off value of 7.0 μV, ΔEAdi had a sensitivity of 100 (92.9–100) % and specificity of 89.5 (78.5–96.0) % for predicting extubation success (Fig. 3B and Table 4). The high diagnostic accuracy of ΔEAdi persists after adjustment of confounders.

**Table 4:** Predictive accuracy of ∆EAdi to predict extubation success in cervical spinal cord injury patients

	AUROC	Sensitivity, %	Specificity, %	Youden index	PPV, %	NPV, %
Crude analysis						
ΔEAdi, μV	0.978 (0.941, 0.995)	100 (92.9, 100)	89.5 (78.5, 96.0)	0.895(0.803, 0.945)	89.3 (79.6, 94.7)	100(94.5, 100)
Adjusted analysis^a^						
ΔEAdi, μV	0.988 (0.963, 0.997)	96.0 (86.3, 99.5)	94.7 (85.4, 98.9)	0.907(0.782, 0.945)	94.1 (84.2, 98.0)	96.4 (87.4, 99.1)

EAdi: diaphragm electrical activity, AUROC: area under receiver operating characteristic, PPV: positive predictive value, NPV: negative predictive value

^a^Adjusted by SOFA, high cervical injury, PaO_2_/FiO_2_, and rapid shallow breathing index

Values for other variables tested for extubation predictability are given in Additional file 1: Table S5. AUROC for ΔEAdi was significantly higher than that for RSBI, baseline VT, ΔVT, baseline NVE and maximal NVE. ΔEAdi resulted in an additive NRI in classifying successful extubation patients over these parameters (Additional file 1: Table S5). Likewise, the decision curve analysis showed that the net benefit of ΔEAdi surpassed that of other predictors (Additional file 1: Fig. S2). More details about the diagnostic performance of mentioned variables are presented in Additional file 1: Table S5 and Fig. S1.

## Discussion

We have proposed a novel method to guide the extubation for CSCI patients. The major finding of this study is that CSCI patients who could be extubated successfully are characterized by increased neural activation of the diaphragm during a “single maximal maneuver”. The absolute increase of EAdi during the maximal effort inspiration is independently associated with successful extubation, and it has a better discriminatory performance for predicting extubation success in CSCI patients compared with the classic approaches.

Although the clinical challenges of respiratory and ventilator management in patients with spinal cord injury are well-appreciated, there are no structured protocols for weaning and extubation attempts in patients with spinal cord injury [22]. The overall extubation rate after attempts was 52.3% in the present study, which was lower than the 75.4% extubation success rate reported in the ATS guideline for weaning in inhomogeneous patients [23]. The result might highlight the need for a specific protocol of extubation and weaning for CSCI patients rather than using traditional approaches to improve the possibility of extubation success or depending on conventional clinical signs to determine extubation outcomes. Higher respiratory rate, RSBI, and increased PaCO_2_ are all classic clinical signs associated with extubation failure [20, 24]. Compared with the traditional predictors, the novel measurement of ΔEAdi during “single maximal maneuver” was found to have more power to discriminate between groups for successful and unsuccessful extubation. These results can potentially modify and improve clinical practice in extubating patients with CSCI. The EAdi monitoring could solve the dilemma of making difficult decisions about extubation in this subset of patients and facilitate the weaning procedure through neurally adjusted ventilatory assistance.

Diaphragm is the primary inspiratory muscle, and impaired diaphragm function is the main reason for extubation failure in CSCI patients [15]. EAdi is correlated with the transdiaphragmatic pressure generated by contraction of diaphragm during inspiration, while the correlation coefficient and the EAdi at quiet breathing exhibit marked variability [25]. It has been reported that changes in EAdi can predict SBT failure and guide ventilator weaning [17]. Hence, we focused on the variation of EAdi during the maximal inspiration in CSCI patients to reflect the preservation of neuromuscular function and guide extubation readiness. Unsurprisingly, we found a higher ΔEAdi during “single maximal maneuver” was independently associated with successful extubation. The specific cut-off point (7.0 μV) of the ΔEAdi provided both high sensitivity and specificity, allowing the identification of patients who could be extubated early.

The relative activation of the diaphragm during SBT was higher in the unsuccessful extubation group, which was probably due to the less motor units caused by spinal cord injury [26]. In our study, the complete motor injury and high cervical injury were more prevalent in patients with unsuccessful extubation. On the condition of reduced number of motor neurons and impaired phrenic nerve conduction, patients may use more of their available capacity to breathe at quiet breathing. In contrast, the increase in EAdi was modest during the maximal inspiration. In addition, the higher NVE during SBT and the single maximal maneuver in the unsuccessful extubation group seems surprising but can be explained. The NVE was calculated as the ratio of tidal volume and EAdi during inspiration. The tidal volume depends on the activation of diaphragm and other inspiratory muscles, and EAdi values are determined by the summation of potential of motor units [16]. In the unsuccessful extubation group, the accessory inspiratory muscles are possibly activated to compensate for the ventilation. However, the less motor units and impaired phrenic nerve conduction could result in a pronounced reduction in EAdi, which in turn results in an elevation of NVE. Inconsistent with previous studies that enrolled patients with intact phrenic nerve conduction [18], our results showed that NVE could not be suitable for predicting extubation outcomes in patients with partial or complete diaphragm denervation.

Our findings are partially consistent with a previous study demonstrating that diaphragm needle EMG [15] and the extent of diaphragm movement assessed via fluoroscopic examination during deep breathing were significantly correlated with ventilator weaning in CSCI patients [27]. Similarly, maximal inspiratory pressure generated from diaphragmatic activity at the time of extubation was independently associated with reintubation [28]. Although there are several methods to evaluate diaphragm function, including ultrasonography, EMG, computerized tomography, magnetic resonance imaging, and video fluoroscopy [29, 30], taking the minimal invasiveness and continuity of monitoring into account, esophageal recordings of EAdi, which have been validated for reliability in healthy subjects and in patients with respiratory dysfunction [18], can be a great tool to guide extubation at the bedside.

Our data confirm the previous results that a subset of patients with high CSCI and respiratory failure can be successfully extubated during acute hospitalization [6, 7]. In the present study, 24/65 (36.9%) of patients with high cervical injury (*C*1–*C*4) were extubated successfully, and 47/65 (72.3%) were weaned from the ventilator, which is consistent with previous reports, showed that almost two-thirds of patients with *C*1–*C*4 injury were weaned before discharge [27, 31]. Since the phrenic nerve originates predominantly from *C*4, with a variable contribution from *C*3 and *C*5, lesions above or within these levels will increase the likelihood of ongoing ventilator dependency. Therefore, these individuals with impaired diaphragm function or complete diaphragm denervation cannot be weaned from mechanical ventilation [10, 32]. The patients in extubation success and unsuccess groups differed in injury level, ISS, and ASIA grades. Still, after multivariable analysis, ΔEAdi remained independently associated with successful weaning.

Of clinical interest, the tracheostomy rate among CSCI patients was 47.7% in this study, consistent with the wide range of 10 to 80% from several studies on spinal cord injury patients. The decision on tracheostomy is greatly influenced by conditions, such as the lesion level and airway complications [1, 11, 12, 14, 33], yet the tracheostomy conversion and timing remain inconclusive [2, 9, 34]. Most studies agree that early tracheostomy in patients with CSCI, typically identified as performed within seven days, was associated with improved outcomes [2, 9]. For instance, early tracheostomy can facilitate ventilator weaning, reduce ICU and hospital length of stay, and enhance patient comfort [9, 35]. Notably, in this study, the median duration of mechanical ventilation before tracheostomy was six days. With these in mind, we believed that identifying CSCI patients who could be expected to fail extubation and thus require prolonged mechanical ventilation would be of substantial clinical importance. To single these patients out for early tracheostomy by ΔEAdi during “single maximal maneuver” will, at the very least, on the one hand, reduce the complications and risks of reintubation and, on the other hand, prevent unnecessary tracheostomy.

Several limitations need to be acknowledged in our study. The main limitation of the study is the nature of the monocentric, non-controlled design. External validation or prospective study may further verify the results. While we conducted the study in a tertiary hospital and the provincial trauma center, the standardized therapy and weaning protocol may have enhanced the credibility of our findings. Second, patients who could not tolerate SBT or had no EAdi wave due to high cervical injury level or complete injury were excluded from our cohort. For those patients, early tracheostomy seems to be more beneficial than extubation attempt. Third, we did not perform airway occlusions during the single maximal maneuver, making it impossible to evaluate the pressure electricity index. Fourth, EAdi monitoring ignores the electrical activity generated by the inspiratory muscles other than the diaphragm, which are often activated in patients with respiratory failure following spinal cord injury to maintain ventilation [36]. Moreover, EAdi surrogates the bilateral action potential while some patients may have asymmetric diaphragm injury which may affect the extubation outcomes.

## Conclusions

In conclusion, increased EAdi during “single maximal maneuver” is independently associated with successful extubation and can help guide early extubation attempts in patients with CSCI. A multicenter prospective study with more participants is necessary before definitive conclusions.

### Supplementary Information

**Additional file 1: Table S1.** Clinical characteristics of included patients. **Table S2.** Comparisons of baseline EAdi and ΔEAdi and ΔVT among patients with different ASIA impairment scales. **Table S3.** Comparisons between patients with or without corresponding sensory impairment. **Table S4.** Multivariable logistics regression exploring the association between ΔEAdi and extubation success. **Table S5.** Predictive performance of possible predictors for extubation success. **Figure S1.** ROC curve analysis of selected variables for predicting extubation success. (A) ΔEAdi; (B) Rapid shallow breathing index; (C) baseline tidal volume; (D) Δ tidal volume; (E) baseline NVE; (F) maximal NVE. **Figure S2.** Decision curve analysis demonstrated the net benefit associated with the use of ΔEAdi over (A) rapid shallow breathing index; (B) baseline tidal volume; (C) ΔVT; (D) baseline NVE; (E) maximal NVE.

## Data Availability

The datasets used and/or analyzed in the current study are available from the corresponding author on reasonable request.

## Abbreviations

ASIA: American Spinal Injury Association
AUROC: Area under receiver operating characteristic curve
CPAP: Continuous positive airway pressure
CSCI: Cervical spinal cord injury
EAdi: Diaphragm electrical activity
ICU: Intensive care unit
ISS: Injury severity score
PaCO2: Partial pressure of arterial carbon dioxide
RSBI: Rapid shadow breathing index
SBT: Spontaneous breathing trial
VT: Tidal volume
