# Supplementary material for: Predicting extubation in patients with traumatic cervical spinal cord injury using the diaphragm electrical activity during a single maximal maneuver
Source: Ann Intensive Care. 2023 Dec 6;13:122. doi: 10.1186/s13613-023-01217-7 (PMC10700269; doi:10.1186/s13613-023-01217-7)
Supplement: Supplementary file 1 — Additional file 1: Table S1. Clinical characteristics of included patients. Table S2. Comparisons of baseline EAdi and ΔEAdi and ΔVT among patients with different ASIA impairment scales. Table S3. Comparisons between patients with or without corresponding sensory impairment. Table S4. Multivariable logistics regression exploring the association between ΔEAdi and extubation success. Table S5. Predictive performance of possible predictors for extubation success. Figure S1. ROC curve analysis of selected variables for predicting extubation success. (A) ΔEAdi; (B) Rapid shallow breathing index; (C) baseline tidal volume; (D) Δ tidal volume; (E) baseline NVE; (F) maximal NVE. Figure S2. Decision curve analysis demonstrated the net benefit associated with the use of ΔEAdi over (A) rapid shallow breathing index; (B) baseline tidal volume; (C) ΔVT; (D) baseline NVE; (E) maximal NVE. [file 13613_2023_1217_MOESM1_ESM.docx]

**Additional file 1**

**Methods**

**Daily screen for the possibility of the initial spontaneous breathing trial (SBT)**

(1) improvement of the underlying condition that required mechanical ventilation, (2) PaO_2_/FiO_2_ ≥ 200 mmHg; PEEP ≤ 5 cm H_2_O FiO_2_ ≤ 50%, and respiratory rate < 35 breaths/min, (3) hemodynamic stability (heart rate < 140 beats/min, no vasopressors required or < 5 μg/kg/min of dopamine or dobutamine, or < 0.2 μg/kg^/^min of norepinephrine), (4) no or low sedation (RASS ≥ -2).

**Criteria for SBT failure**

SBT failure was defined if any of the following criteria is reached: (1) hypoxemia or hypercapnia: SpO_2_ <90% or PaO_2_ <60 mmHg, or increase in PaCO_2_ >10 mmHg, (2) hemodynamic instability: heart rate changed >20%; systolic blood pressure (SBP) > 180 or < 90 mmHg; SBP changed > 20%, newly onset severe arrhythmias, increment of vasopressors, (3) 8 breaths/min > respiratory rate >35 breaths/min, tidal volume < 4 mL/kg, (4) somnolence, coma, agitation, anxiety, diaphoresis, and other onset or worsening of discomfort deemed by the clinical team, resumed mechanical ventilation. Patients who did not complete the initial SBT were switched to the previous ventilation settings and screened 24 hours later.

**Assessment for cuff leak and cough strength**

When performing the cuff leak test, the ventilator was switched to volume assist-control mode with tidal volume set at 10-12mL/kg of predicted body weight, and after the suction of oral secretions, the cuff was deflated. Four to six consecutive breaths were used to calculate the difference in expiratory tidal volume before and after the cuff deflation [1]. The cutoff values were 110 mL in volume and 15% in proportion [2]. For patients who failed the cuff leak test, but are otherwise ready for extubation, systemic steroids were prescribed.

The cough was evaluated by placing a white card about 1.5 cm away from the end of the endotracheal tube and asking the patient to cough (3 to 4 times). Adequate cough was considered if wetness appeared on the card. Patient who succeeded the initial SBT and cut leak test but without sufficient cough strength were switched to the previous ventilation settings and screened 24 hours later.

**Local protocols for post-extubation non-invasive respiratory support**

Post-extubation non-invasive respiratory support (NIRS) is applied if: (1) Hypercapnia: increase in PaCO_2_ >10 mmHg or pH <7.35 for patients with COPD, (2) Hypoxemia: SpO_2_ < 90%. Noninvasive ventilation was started at an inspiratory positive airway pressure of 5 cmH_2_O, increasing to a maximum inspiratory pressure of 20 cmH_2_O, aiming to achieve an expiratory tidal volume between 6 and 8 mL/kg of predicted body weight and a respiratory rate lower than 30 breaths/min. PEEP and inspired oxygen fraction were titrated to maintain SpO_2_ of at least 90%. High-flow oxygen therapy was primarily applied in patients with hypoxemic respiratory failure to maintain SpO_2_ of at least 90%. Prophylactic NIRS was not applied.

**Local protocols for reintubation.**

The following criteria were used for reintubation: loss of consciousness or psychomotor agitation hindering nursing care and requiring sedation, severe hemodynamic instability (defined by systolic arterial blood pressure below 90 mmHg or mean arterial blood pressure below 65 mmHg) despite fluid resuscitation or need for vasopressors, respiratory or cardiac arrest, massive aspiration, persistent inability to clear respiratory secretions, heart rate of fewer than 50 beats/min with loss of alertness, or two of the following criteria: frank worsening of respiratory distress under NIV, SpO_2_ remaining below 90% despite FiO_2_ 100%, respiratory rate > 40 breaths/min, or pH <7.25.

**Diagnostic criteria of VAP**

Ventilator-associated pneumonia (VAP) was defined as pneumonia that arises at least 48 hours after endotracheal intubation or less than 48 hours after extubation. Diagnosis of pneumonia should meet the following criteria [3]: new or progressive infiltrates on chest radiograph; at least two or more of the following clinical findings suggesting infection: new onset of fever, oral temperature T>38.3°C or T<36°C; purulent sputum; WBC > 12×10^9^/L or < 4×10^9^/L, or bands >10%.

**Selection of variables in multivariable analysis**

Multivariable logistics regression was used to evaluate the association between ΔEAdi and extubation success. According to published studies investigating factors associated with extubation outcomes, and clinical relevance [4, 5], we originally chosen variables including RSBI, respiratory rate, baseline tidal volume, SOFA, ISS, age, PaO_2_/FiO_2_, PaCO_2_, level of injury. Since RSBI, respiratory rate and tidal volume were mathematically coupled, we included the most commonly studied RSBI. The ISS score reflects the severity of injuries at admission, while as a component of ISS score, the cervical injury was more associated with both respiratory drive and extubation failure compared with the total ISS score. Additionally, the AIS score of chest and brain were comparable between groups, and we finally included cervical spinal cord injury rather than ISS into the regression model. Lower respiratory drive (EAdi) could result in elevated PaCO_2_, which in turn results in extubation failure. Hence, PaCO_2_ would partially mediate the correlation between EAdi and extubation outcomes, and was not included in the analysis [6]. Age was comparable between groups. With 50 patients succeeded the first extubation, we selected five variables (∆EAdi, SOFA, RSBI, C1-C4 injury, PaO_2_/FiO_2_) in accordance with the principle of ten events per variable [7]. The variance inflation factor (VIF) suggested no multicollinearity.

**Reference**

1. Jaber S, Chanques G, Matecki S, Ramonatxo M, Vergne C, Souche B, et al. Post-extubation stridor in intensive care unit patients. Risk factors evaluation and importance of the cuff-leak test. Intensive Care Med. 2003;29(1):69-74.

2. Kuriyama A, Jackson JL, Kamei J. Performance of the cuff leak test in adults in predicting post-extubation airway complications: a systematic review and meta-analysis. Crit Care. 2020;24(1):640.

3. Torres A, Niederman MS, Chastre J, Ewig S, Fernandez-Vandellos P, Hanberger H, et al. International ERS/ESICM/ESCMID/ALAT guidelines for the management of hospital-acquired pneumonia and ventilator-associated pneumonia: Guidelines for the management of hospital-acquired pneumonia (HAP)/ventilator-associated pneumonia (VAP) of the European Respiratory Society (ERS), European Society of Intensive Care Medicine (ESICM), European Society of Clinical Microbiology and Infectious Diseases (ESCMID) and Asociacion Latinoamericana del Torax (ALAT). Eur Respir J. 2017;50(3):1700582.

4. Baptistella AR, Sarmento FJ, da Silva KR, Baptistella SF, Taglietti M, Zuquello RA, et al. Predictive factors of weaning from mechanical ventilation and extubation outcome: A systematic review. J Crit Care. 2018;48:56-62.

5. Schreiber AF, Garlasco J, Vieira F, Lau YH, Stavi D, Lightfoot D, et al. Separation from mechanical ventilation and survival after spinal cord injury: a systematic review and meta-analysis. Ann Intensive Care. 2021;11(1):149.

6. Lederer DJ, Bell SC, Branson RD, Chalmers JD, Marshall R, Maslove DM, et al. Control of Confounding and Reporting of Results in Causal Inference Studies. Guidance for Authors from Editors of Respiratory, Sleep, and Critical Care Journals. Ann Am Thorac Soc. 2019;16(1):22-8.

7. Riley RD, Ensor J, Snell KIE, Harrell FE, Jr., Martin GP, Reitsma JB, et al. Calculating the sample size required for developing a clinical prediction model. BMJ. 2020;368:m441.

Table S1 Clinical characteristics of included patients.

|  | Overall (n=107) | Successful Extubation (n=50) | Unsuccessful Extubation (n=57) | *p* value |
| --- | --- | --- | --- | --- |
| Height, cm | 174 [168, 175] | 171 [165, 175] | 175 [170, 175] | 0.034 |
| Body weight, kg | 70 [65, 76] | 70 [65, 78] | 70 [65, 76] | 0.997 |
| Predicted body weight, kg | 66 [61, 71] | 66 [61, 69] | 66 [64, 71] | 0.064 |
| Additional injuries, n (%) | | | | |
| Craniocerebral | 46 (43.0) | 21 (42.0) | 25 (43.9) | 1.000 |
| Chest | 36 (33.6) | 8 (16.0) | 28 (49.1) | 0.001 |
| Abdomen | 9 (8.4) | 2 (4.0) | 7 (12.3) | 0.234 |
| Limbs | 19 (17.8) | 6 (12.0) | 13 (22.8) | 0.228 |
| AIS of neck and head | 3 [3, 4] | 3 [3, 4] | 4 [3, 4] | 0.218 |
| AIS of chest | 0 [0, 2] | 0 [0, 1] | 1 [0, 2] | 0.093 |
| Respiratory parameters during CPAP | | | | |
| Baseline VT, mL | 302 [217, 360] | 342 [296, 375] | 223 [187, 325] | <0.001 |
| Maximal VT, mL | 601 [330, 750] | 716 [639, 866] | 334 [243, 590] | <0.001 |
| ΔVT, mL | 279 [118, 379] | 379 [298, 502] | 122 [73, 232] | <0.001 |
| FiO_2_ | 0.4 [0.4, 0.4] | 0.4 [0.4, 0.4] | 0.4 [0.4, 0.4] | 0.820 |
| PaO_2_, mmHg | 116.9 [95.2, 142.6] | 133.9 [106.0, 154.8] | 110.3 [88.8, 125.3] | <0.001 |
| Respiratory parameters during PSV | | | | |
| VT, mL | 424 [388, 460] | 441 [399, 482] | 415 [384, 454] | 0.049 |
| VT, mL/kg | 6.4 [5.8, 7.6] | 7.0 [6.1, 7.8] | 6.3 [5.7, 7.3] | 0.102 |
| Pressure support, cmH2O | 10 [8,12] | 10 [8,12] | 10 [8,13] | 0.493 |
| PEEP, cmH2O | 5 [5, 5] | 5 [5, 5] | 5 [5, 6] | 0.001 |
| Respiratory rate, | 18 [15, 21] | 16 [15,19] | 20 [16, 22] | <0.001 |
| RSBI | 42 [33, 54] | 35 [31, 46] | 47 [37, 60] | <0.001 |

AIS, Association Impairment Scale; FiO_2_, fraction of inspired oxygen; CPAP, continuous positive airway pressure; PSV, pressure support ventilation; PEEP, positive end expiratory pressure;VT, tidal volume; RSBI, rapid shallow breathing index.

Table S2 Comparisons of baseline EAdi and ΔEAdi among patients with different ASIA impairment scales.

|  | Baseline EAdi, μV | ΔEAdi, μV |
| --- | --- | --- |
| ASIA impairment scale |  |  |
| A (n=16) | 5.0 [3.4, 8.6] | 4.1 [2.4, 15.6] |
| B (n=32) | 4.9 [3.1, 5.9] | 3.8 [1.7, 9.4] |
| C (n=49) | 6.5 [4.9, 8.3] | 10.9 [5.0, 16.1] |
| D (n=10) | 7.6 [6.8, 8.0] | 15.5 [9.8, 19.4] |
| *p* value | 0.012 | < 0.001 |
| Complete motor injury |  |  |
| A+B (n=48) | 4.9 [3.3, 6.7] | 3.8 [2.0, 9.9] |
| C+D (n=59) | 7.0 [5.2, 8.2] | 11.9 [5.4, 16.8] |
| *p* value | 0.006 | < 0.001 |

EAdi, diaphragm electrical activity; ASIA, American Spinal Injury Association; ASIA A, absence of sensory and motor function; ASIA B, sensory but not motor function is preserved; ASIA C, motor function is preserved with key muscles have a muscle grade less than 3; ASIA D, motor function is preserved with key muscles have a muscle grade greater than or equal to 3.

Table S3 Comparisons between patients with or without corresponding sensory impairment.

|  | Sensory injury (n=26) | No sensory injury (n=81) | *p* value |
| --- | --- | --- | --- |
| Baseline EAdi, μV | 5.4 [3.5, 7.1] | 6.0 [4.2, 8.0] | 0.187 |
| ∆EAdi, μV | 7.3 [2.2, 14.3] | 8.1 [3.0, 15.8] | 0.443 |
| Baseline VT, mL/kg | 4.7 [2.8, 5.5] | 4.6 [3.3, 5.6] | 0.490 |
| Extubation success, n (%) | 11 (42.3) | 39 (48.1) | 0.769 |

EAdi, diaphragm electrical activity; VT, tidal volume.

Table S4 Multivariable logistics regression exploring the association between ΔEAdi and extubation success.

| Variables | Adjusted Odds ratio | 95% CI | *p* value | VIF |
| --- | --- | --- | --- | --- |
| ΔEAdi | 2.03 | 1.52-3.17 | <0.001 | 1.7 |
| SOFA | 0.65 | 0.41-0.89 | 0.027 | 1.4 |
| C1-C4 injury | 0.40 | 0.05-2.89 | 0.363 | 1.1 |
| PaO_2_/FiO_2_ | 1.01 | 0.99-1.03 | 0.256 | 1.1 |
| RSBI | 1.00 | 0.97-1.03 | 0.853 | 1.3 |

EAdi, diaphragm electrical activity; FiO2, fraction of inspired oxygen; RSBI, rapid shallow breathing index; SOFA, sequential organ failure assessment.

Table S5 Diagnostic accuracy of possible predictors for extubation success.

| Variables | AUROC (95%CI) | Cutoff | Sensitivity, % | Specificity, % | PPV, % | NPV, % | p value^*^ | NRI, % ^§^ |
| --- | --- | --- | --- | --- | --- | --- | --- | --- |
| ΔEAdi, μV | 0.978 (0.941, 0.995) | 7.0 | 100 (92.9, 100) | 89.5 (78.5, 96.0) | 89.3 (79.6, 94.7) | 100 (94.5, 100) | - | - |
| RSBI | 0.774 (0.666, 0.855) | 121 | 98.0 (89.4, 99.9) | 57.9 (44.1, 70.9) | 67.1 (60.0, 73.5) | 97.1 (82.4, 99.6) | <0.001 | 48.4 (35.1, 63.4) |
| Baseline VT, mL/kg | 0.790 (0.692, 0.868) | 3.6 | 100 (92.9, 100) | 61.4 (47.6, 74.0) | 69.4 (62.1, 75.9) | 100 (91.7, 100) | <0.001 | 59.6 (45.2, 81.6) |
| ΔVT, mL/kg PBW | 0.934 (0.880, 0.969) | 4.1 | 90.0 (78.2, 96.7) | 87.7 (76.3, 94.9) | 86.5 (76.1, 92.8) | 90.9 (81.2, 95.9) | 0.006 | 28.8 (14.8, 42.4) |
| Baseline NVE, mL/μV | 0.742 (0.630, 0.827) | 57.6 | 84.0 (70.9, 92.8) | 59.7 (45.8, 72.4) | 64.6 (56.6, 71.9) | 81.0 (68.5, 89.3) | <0.001 | 78.9 (62.9, 97.5) |
| Maximal NVE, mL/μV | 0.860 (0.767, 0.919) | 39.1 | 80.0 (66.3, 90.0) | 82.5 (70.1, 91.3) | 80.0 (69.1, 87.7) | 82.5 (72.7, 89.2) | 0.001 | 42.6 (27.8, 60.2) |

EAdi, diaphragm electrical activity; PBW, predicted body weight; VT, tidal volume; NVE, neuroventilatory efficiency; RSBI, rapid shallow breathing index; AUROC, area under receiver operating characteristic; PPV, positive predictive value; NPV, negative predictive value; NRI, net reclassification index

* denotes comparisons of AUROC between ΔEAdi and other parameters.

§ denotes additive NRI with 95%CI of ΔEAdi over other parameters.


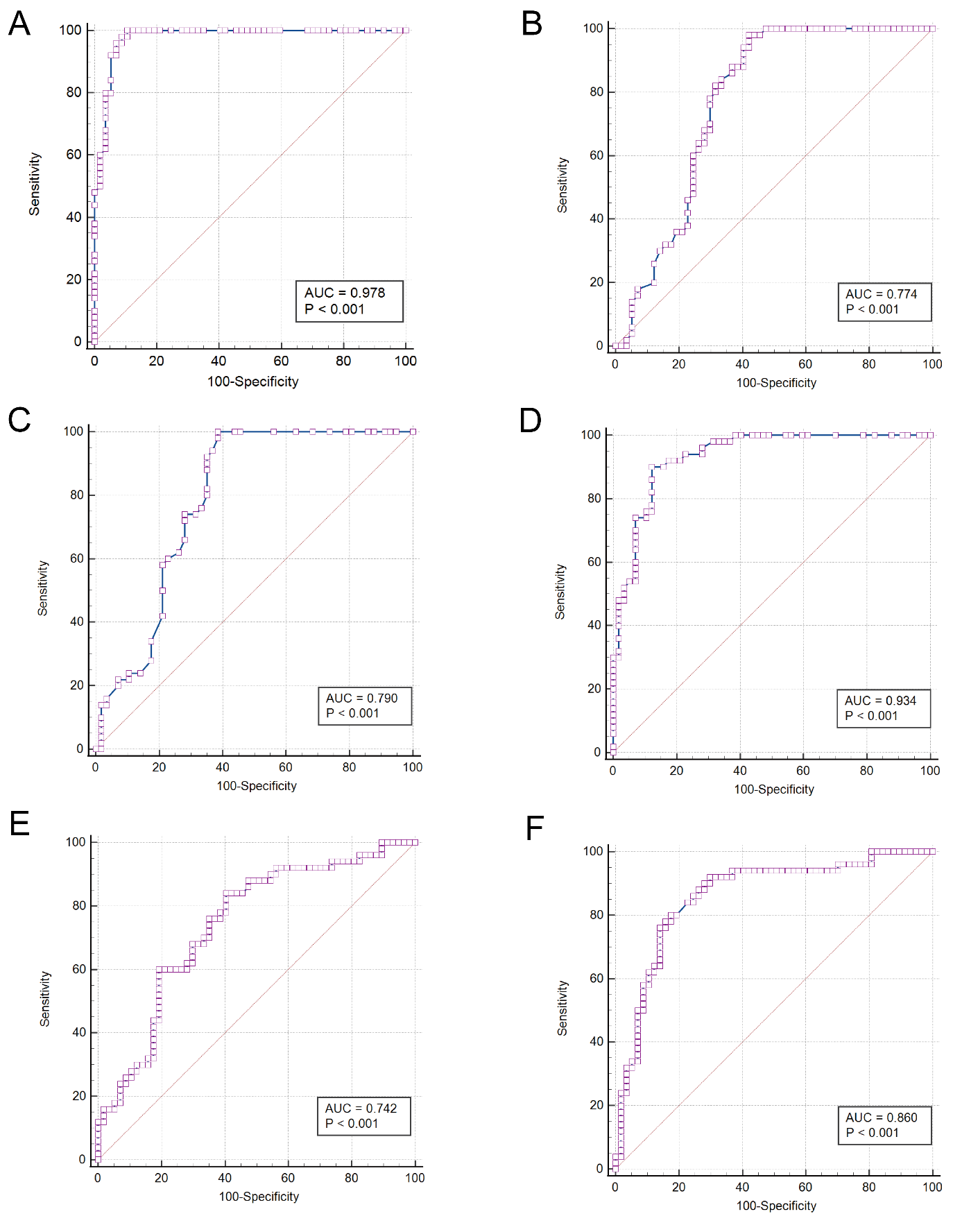


Figure S1 ROC curve analysis of selected variables for predicting extubation success. (A) ΔEAdi; (B) Rapid shallow breathing index; (C) baseline tidal volume; (D) Δ tidal volume; (E) baseline NVE; (F) maximal NVE.


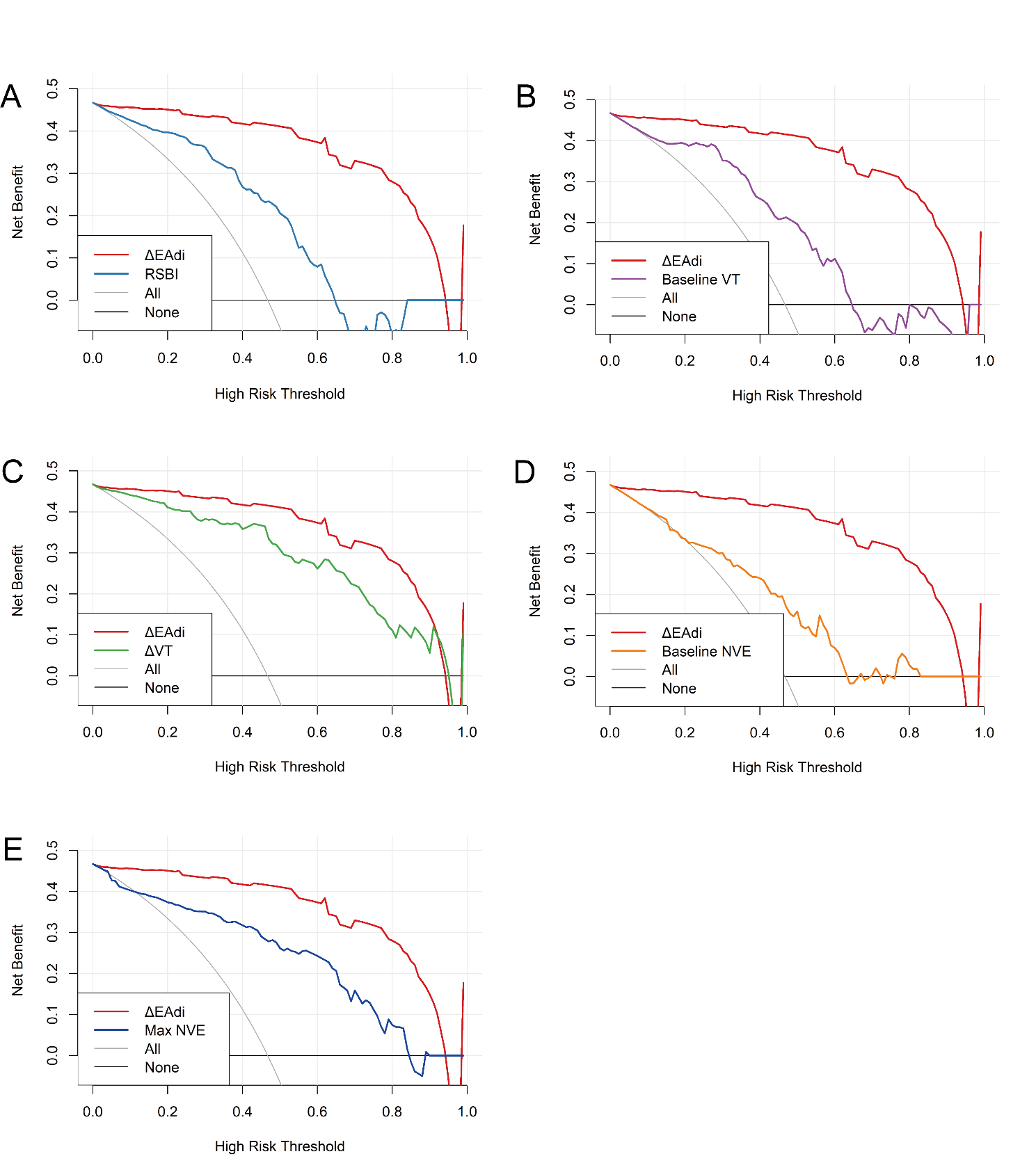


Figure S2 Decision curve analysis demonstrated the net benefit associated with the use of ΔEAdi over (A) rapid shallow breathing index; (B) baseline tidal volume; (C) Δ tidal volume; (D) baseline NVE; (E) maximal NVE.
